# A new era for research into aging

**DOI:** 10.7554/eLife.65286

**Published:** 2021-01-28

**Authors:** Matt Kaeberlein, Jessica K Tyler

**Affiliations:** 1 Department of Laboratory Medicine and Pathology, University of WashingtonSeattleUnited States; 2 Department of Pathology and Laboratory Medicine, Weill Cornell MedicineNew YorkUnited States

**Keywords:** aging, geroscience, longevity

## Abstract

eLife is publishing a special issue on aging, geroscience and longevity to mark the rapid progress made in this field over the past decade, both in terms of mechanistic understanding and translational approaches that are poised to have clinical impact on age-related diseases.

Every major cause of death and disability in the developed world shares a greatest risk factor, and it is probably not what most people would think. Smoking, obesity, a sedentary lifestyle and drinking too much alcohol all contribute to disease: however, their contributions are small in comparison to the physiological changes that result from aging. Whether biological aging causes the many functional declines that occur with age, or just permits them, is perhaps open for debate, but there is no question that, for most of us, biological aging determines how and when we and our loved ones will get sick and die.

This connection between aging and disease has become particularly consequential during the COVID-19 pandemic, with the vast majority of severe cases and deaths occurring among the elderly. Given this obvious relationship, it is somewhat surprising how slowly the biomedical research community has come to appreciate the importance of biological aging in many of the disease processes under study. It is our hope that the articles in the eLife special issue on aging, geroscience and longevity will contribute to a greater appreciation and understanding of aging biology among the broader scientific community. A number of the authors of these articles also spoke at a recent eLife symposium on this topic.

Today, unfortunately, too many scientists study individual diseases without recognizing the impact of aging biology. It is still common, for example, to see research studies in cancer, neuroscience, metabolism and other fields where young animal models (such as 4–6 month old mice) are used to study disease processes that almost exclusively occur in old people. ‘Mice are not people’ is a standard refrain when explaining why so many preclinical therapies fail in human trials. Perhaps the mouse isn’t the problem. Failing to account for the physiological changes that occur during aging, both in mice and in people, may be a much bigger reason why so much preclinical research fails to translate to the clinic.

It is still common to see research studies in cancer, neuroscience, metabolism and other fields where young animal models are used to study disease processes that almost exclusively occur in old people.

Thinking about certain conserved molecular mechanisms as 'hallmarks' or 'pillars' of aging (Kennedy et al., 2014; López-Otín et al., 2013) has benefitted researchers within the field, and has also allowed scientists outside the field to begin to recognize how aging biology impacts on their own research. Many of these conserved mechanisms are studied in the papers in this special issue, including telomere attrition, mitochondrial dysfunction, cellular senescence, epigenetic alterations, stem cell exhaustion, genomic instability, and loss of proteostasis.

Another important advance in aging research has been the development of a concept called geroscience: researchers in this area seek to understand mechanistically how the hallmarks of aging cause age-related disease and functional decline (Sierra and Kohanski, 2017). The growth of the geroscience concept also reflects a recognition that aging research is much closer to clinical application than it was twenty years ago. Numerous interventions have been developed that target one or more of the hallmarks of aging in order to delay, or even reverse, age-related functional declines. In rodents, for example, it has been shown that the drug rapamycin can prevent age-related diseases and improve function in multiple aged tissues and organs. Now, in the eLife special issue on aging, An et al. report that rapamycin also works in the oral cavity and can reverse periodontal disease in mice (An et al., 2020). Other articles suggest translational strategies to target specific hallmarks of aging for intervertebral disc degeneration (Cherif et al., 2020) and age-related heart disease (Chiao et al., 2020). At the time of writing there are two review articles and more than 20 research articles in the special issue, and more will be added over time.

The future of aging research is brighter than ever before, and the pace of discovery is only increasing. We look forward to major breakthroughs over the next few years that will revolutionize the way we think about aging biology and have the potential to significantly impact human healthspan and longevity.
